# Effectiveness and Safety of Oral Propranolol versus Other Treatments for Infantile Hemangiomas: A Meta-Analysis

**DOI:** 10.1371/journal.pone.0138100

**Published:** 2015-09-16

**Authors:** Xiaohan Liu, Xinhua Qu, Jiawei Zheng, Ling Zhang

**Affiliations:** 1 College of Stomatology, Shanghai Jiao Tong University School of Medicine, Shanghai, China; 2 Shanghai Key Laboratory of Orthopedic Implant, Shanghai Ninth People’s Hospital, Shanghai Jiao Tong University School of Medicine, Shanghai, China; 3 Department of Oral and Maxillofacial Surgery, Shanghai Ninth People’s Hospital, Shanghai Jiao Tong University School of Medicine, Shanghai, China; Emory University, UNITED STATES

## Abstract

**Background:**

Epidemiological studies evaluating treatments for infantile hemangiomas have produced inconsistent results. A meta-analysis of published data was conducted to investigate the effectiveness and safety of oral propranolol versus other treatments for infantile hemangiomas.

**Methods:**

A meta-analysis was conducted based on literature (published from 1960 to December 1, 2014) found on the PubMed, EMBASE, and OVID search engines. Pooled odds ratios (ORs) and 95% confidence intervals (CIs) were estimated for the outcome measures. Heterogeneity, publication bias and subgroup analysis were performed.

**Results:**

A total of 61 studies involving 5,130 participants met the inclusion criteria. Propranolol was found to be a more effective modality in treating IHs (ORs = 0.92; 95%CI, 0.89–0.95) and had fewer complications compared to the other treatments including systemic steroids (ORs = 0.68; 95% CI, 0.59–0.76); laser ablation (ORs = 0.55; 95% CI, 0.43–0.67); other beta-adrenergic blockers (ORs = 0.56; 95% CI, 0.50–0.61) and surgery (ORs = 0.55; 95% CI, 0.28–0.81). A subgroup analysis of propranolol showed that a dose of 2 mg/kg/day or more yielded better outcomes (ORs = 0.92; 95% CI, 0.88–0.95; ORs = 0.95; 95% CI, 0.89–1.00), and IHs that had not been previously treated had better responses to propranolol treatment (ORs = 0.95; 95% CI, 0.91–0.98).

**Conclusions:**

The meta-analysis demonstrated that propranolol was more effective and safer than other therapies in treating IHs. It provides strong evidence for supporting the use of propranolol as a first-line therapy for IHs.

## Introduction

Infantile hemangiomas (IHs) are the most common type of benign tumor, affecting approximately 10% of children [1]. Although, most IHs have a self-limiting course, some may result in residual telangiectasias or redundant skin. Therefore, early intervention is indicated for IHs [2].

Systemic corticosteroids used to be the first-line treatment for IHs. However, long term use tends to result in serious side effects such as hypertension, adrenal cortical insufficiency, and delayed of growth [3].

Other treatment modalities including laser ablation, interferon-á, vincristine and surgical excision are reserved as second- or third-line therapy for IHs because of their inconsistent efficacy, multiple complications and potential toxicity [4].

In 2008, propranolol, a nonselective beta-blocker, was serendipitously discovered to be effective for treating IHs. Leaute-Lamberer et al. successfully treated 11 children with oral propranolol and observed tumor color regression in all cases soon after the treatment. Since then, large clinical studies have confirmed the efficacy and safety of propranolol [5].

Recently, other nonselective beta- blockers such as atenolol and timolol have also been found to be useful in treating IHs [6].

The aim of this meta-analysis was to systematically review the existing published data regarding the treatment of IHs, and to compare the effectiveness and safety of propranolol with other therapies. A subgroup analysis was also performed to evaluate the relationship between the effectiveness of propranolol and factors including location, dosage and previous treatment.

## Materials and Methods

The study protocol was in accordance with the PRISMA guidelines (S1 PRISMA Checklist) [7].

### Search strategy

A literature search was performed by searching the PubMed, EMBASE, and OVID databases through December 2014. Combinations of the following terms were used in the search (1) outcome terms: hemangiomas, infantile hemangiomas and complicated hemangiomas; and (2) therapeutic terms: propranolol, systemic steroids, beta-blocker, laser ablation, vincristine, and surgical intervention. The review articles were assessed for relevant references.

### Selection criteria

The studies were evaluated by two independent reviewers (XHL and XHQ). To avoid bias, discrepancies were resolved by a third reviewer (JWZ) through a discussion. To avoid the issue of missing data in certain studies, the respective authors were contacted and asked to provide relevant information.

Studies that met the following criteria were included in the meta-analysis: (1) infantile population; (2) study sample size≧20(the timolol/atenolol sample size was≧10); (3) retrospective studies, prospective studies or RCT; (4) clear description of the therapy (propranolol, systemic steroids, laser ablation, etc.); and (5) well-reported outcome measures (including explicit reporting of the response rate). The studies that did not meet the inclusion criteria were excluded during the initial review.

### Data extraction and quality assessment

Two reviewers (XHL and XHQ) independently extracted the data based on a standard data collection form. A third reviewer (JWZ) resolved any discrepancies by discussing and consulting on the original articles. For each identified study, the following data were collected: last name of the first author, publication year, country, study design, number of cases, participants’ sex and age, location of the IHs, previous treatments, dosage of treatment, response rate and complications.

### Data synthesis and statistical analysis

Odds ratios (ORs) and 95% CIs that reflected a degree of control for potential confounders were extracted from the selected studies for analysis [8]. In this meta-analysis, either a random-effects model (DerSimonian-Laird method) or a fixed-effects model (Mantel-Haenszel method) was used for analysis. Heterogeneity among the studies was evaluted by using *I* ^*2*^ statistics. *I* ^*2*^ values of 25%, 50% and 75% were defined as low, moderate, and high, respectively [9]. A subgroup analysis was conducted to identify associations between the efficiency of propranolol and relevant study characteristics (location of IHs, geographical location of patients, mean dosage of treatment and prior therapy). Funnel plot asymmetry measured by Egger’s and Begg’s tests, was used to assess publication bias [10, 11]. Probability values <0.05 were considered statistically significant [12]. Data analysis was performed using R software 2.13.0, package (meta package metaprop and forest functions).

## Results

### Eligible studies and study characteristics

**Fig 1:**
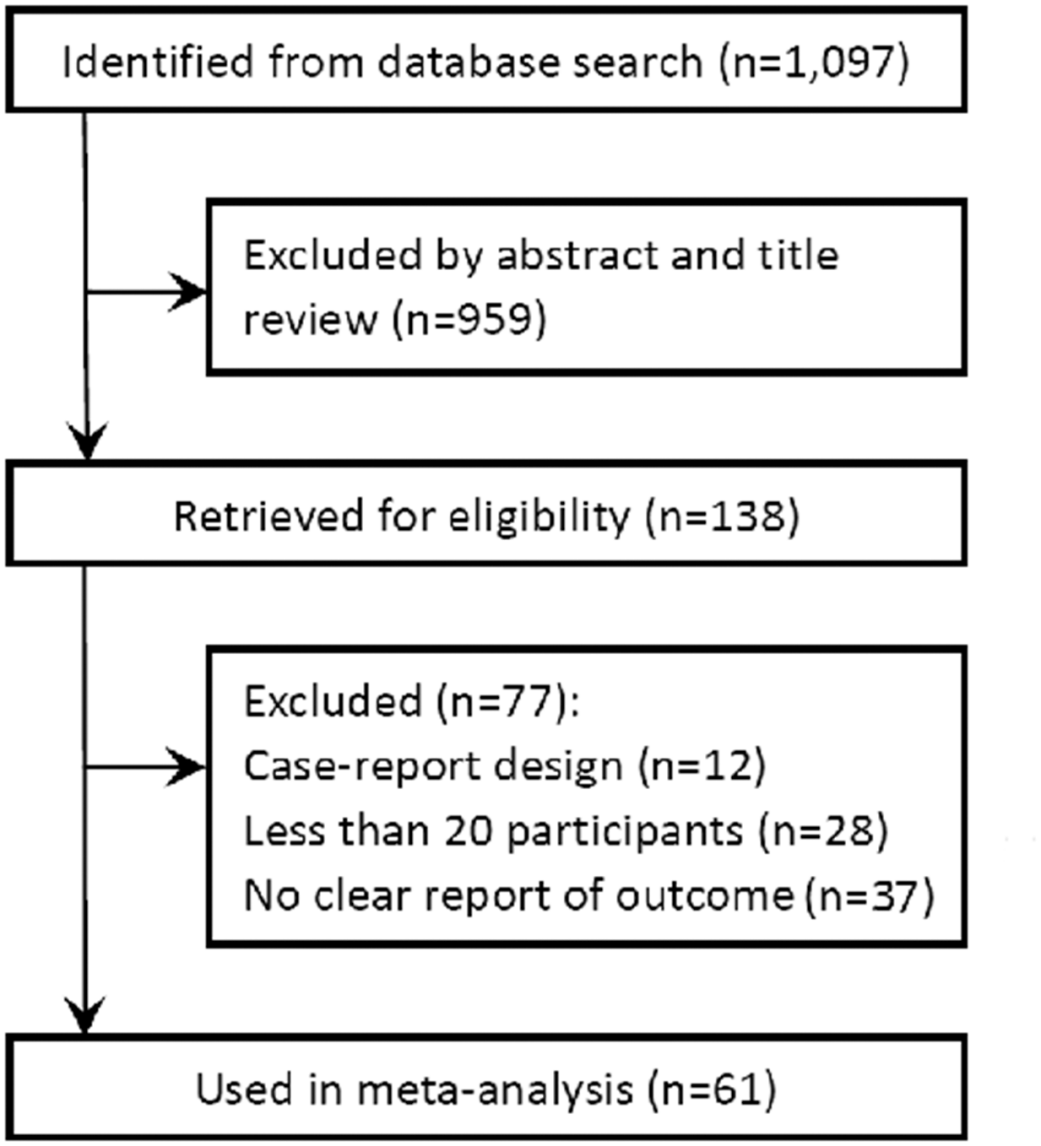
Flow chart of the study selection process.

A total of 61 studies [13–73] were selected from 1097 potential articles for the meta-analysis (Fig 1). The characteristics of the selected articles are listed in Tables 1 and 2. The analysis included 5,130 IH cases from the 61 studies; of these cases, 3761 were located in the head and neck, 216 were located in the trunk, and 160 were located in the extremities. Of the included studies, 30 studies chose propranolol as the definitive treatment; 31 studies used other treatments (15 studies used systemic steroids, 7 studies used laser ablation, 2 studies used surgery, 3 studies used atenolol and 4 studies used timolol). The average age of the patients was 6.2 months. Evaluation of the outcomes was based on visual measurements, photograph scoring, Doppler ultrasonography or MRI.

**Table 1:** Characteristics of studies that used propranolol for treating IHs.

Study (propranolol)	Study design	Number of patients /Sex (M:F)	Location/Previous therapy	Age(m)/Dose(mg/kg/day)	Number of response	Complications
Sans et al.2009/America [13]	RS	32/11:21	H&N21;Torso3;Extremity2;Multiple6/Yes	4.2/2	32	Agitation2;Asthma1;Cold-extremity1;Insomnia2;Others3
Buckmiller et al.2010/America [14]	RS	32/5:27	H&N22;Multiple 10/Yes	4.9/2	16	Allergy1;Asthma1;Gastroesophagealreflux2;Fatigue6
Holmes et al.2010/ Britain [15]	RS	31/NR	NR/No	NR/3	31	None
Schupp et al.2011/German [23]	RS	55/15:40	H&N42;Multiple 13/Yes	6.4/2	54	Asthma2;Cold-extremity6;Gastroenteropathy2;Fatigue4;Others3
Fuchsman et al.2011/America [18]	RS	39/12:27	H&N39/Yes	4.1/2	37	Insomnia5
Schiestl et al.2011/Europe [22]	RS	25/9:16	H&N25/Yes	3.6/2	25	Hypotension6
Hogeling et al.2011/America [19]	RCT	20/7:13	H&N17;Torso1;Extremity1;Multiple1/Yes	2.25/2	16	Bronchiolitis4;Cold-extremity1;Gastroenteropathy1;Infection2;Insomnia2;Ulceration1;Others2
Zvulunov et al.2011/Israel [25]	RS	42/5:37	NR/No	28/2.1	42	Dyspnea1;Insomnia2;Somnolence1
Cushing et al.2011/America [16]	RS	44/9:35	H&N44/Yes	5.8/2	39	None
Jin et al.2011/China [20]	RS	78/NR	NR/No	3.7/2	77	Insomnia12
Zaher et al.2011/Europe [24]	RS	30/NR	H&N30/No	NR/2	29	None
Graaf et al.2011/Netherlands [17]	RS	28/7:21	H&N28/Yes	8.8/2.2	28	Cold-extremity3;Constipation3;Hyperreactivity3;Hypoglycemia2;Hypotension16;Insomnia8
Chai et al. 2014/China [36]	RS	27/6:21	H&N22;Torso5/No	4.1/2	27	somnolence7
Price et al. 2011/America [21]	RS	68/NR	NR/No	4.5/2	56	Hypoglycemia1;Skin rash2
Rössler et al.2011/German [29]	RS	30/NR	NR/No	4.5/2	25	Diarrhea2;Hypotonia3;Reducedactivity3
Meng et al.2012/China [28]	RS	22/9:13	H&N22/Yes	5.5/1.5	20	Diarrhea2;Hypotension5
Lv et al.2012/China [27]	RS	37/10:27	H&N37/Yes	2.8/2	26	Diarrhea9;Nausea1
Laranjo et al.2014/Portugal [38]	RS	30/15:15	H&N21;Torso5;Extremity4/No	6/2.8	30	None
Graaf et al.2013/Portugal [30]	RS	28/NR	NR/No	6.8/2	28	Bronchospasm4;Constipation3;Hypoglycaemiae2;Hypotension1; Sleep-disturban11
Ma et al.2013/German [31]	RS	89/37:52	H&N51;Torso24;Extremity8;Perineum6/No	3.56/0.75	65	Cold-extremity1;Diarrhea3;Hypoglycemia4;Insomnia2;Nusea2
Georgountzou et al.2012/Greece [26]	RS	28/8:20	H&N4;Multiple4/Yes	5.59/2	21	Hypotension4
Mcswiney et al.2014/German [39]	RS	20/5:15	H&N19;Torso1/No	6/2	20	Cold-extremity1
Sondhi et al.2013/America [33]	RS	31/9:22	H&N14;Torso8;Etremity4;Multiple5/Yes	5/2	28	Bronchospasm1;Insomnia2
Vercellino et al.2013/Italy [34]	RS	68/19:49	H&N59;Torso3;Extremity2;Viscera4/Yes	12.6/1.6	63	None
Sadykov et al.2013/German [32]	RS	71/15:56	H&N71/Yes	5.8/2	42	Others20
Szychta et al.2014/Britain [41]	RS	60/NR	H&N55;Torso2;Extremity3/No	4.06/3.71	37	Diarrhea3;Hypotension1;Sleep-disturban1;Rash1
Xiao et al.2013/China [35]	RS	64/13:51	H&N52;Torso6;Extremity6/Yes	3.3/2	59	Bradycardia1;Bronchiolitis1;Cold-Extemity2;Constipation2;Diarrhea4;Insomnia3;Gastroenteropathy6
Hassan et al.2014/Egypt [37]	RS	30/9:21	Head&Neck19;Torso8;Extremity3/No	3.7/1.5	30	Cold-extrmity1;Constipation2;Hypoglycemia1;Tachypnea2
Luo et al.2014/China [42]	RS	635/204:431	NR/No	0.57/2	579	Bradycardia2;Diarrhea3;Hyperkalemia4;Emaciation3
Sagi et al.2014/Israel [40]	RS	99/19:80	H&N80;Multiple19/No	0.3/2	98	Dyspnea2;Nausea1;Insomnia29

NR, not reported; RS, retrospective study; PS, prospective study; H&N, head and neck

**Table 2:** Characteristics of studies that used other therapies for treating IHs.

**Study (Systemic Steroids)**	**Study design**	**Number of patients /Sex (M:F)**	**Location/Previous therapy**	**Age(m)/Dose(mg/kg/day)**	**Number of response**	**Complications**
Kushner et al.1979/Japan [43]	RS	25/NR	H&N25/No	4.2/2	21	NR
Narcy et al.1985/America [44]	RS	21/NR	H&N21/No	NR/2	7	NR
Chowdri et al.1994/America [45]	RS	74/NR	H&N48;Torso11;Extremity15/No	36/10	32	Cushingoid-apperance2
Sadan et al.1996/Israel [46]	RS	60/15:45	H&N60/No	5.5/3.5	56	Growth-retardation1;Moon-face32;Osteoporosis1
Blei et al.1999/Europe [47]	RS	30/NR	H&N27;Extremity3/No	NR/3.5	8	Endocrine-disorder4;Growth-retardation3;Moon-face7
Chen et al.2000/China [48]	RS	155/NR	H&N155/No	3.8/10	93	Cushingoid-apperance2;Cutaneous-diseases5
Jalil et al.2006/America [49]	RCT	50/NR	NR/No	NR/2	19	Overall,22%
Pope et al.2007/America [50]	RCT	20/3:17	H&N20/No	3/2	8	Endocrine-disorder16;Hypertensions4
Chantharatanapiboon et al.2008/Thailand [51]	RS	160/49:111	H&N134;Extremity26/No	5.5/1.5	144	NR
Rössler et al.2008/German [52]	RS	38/11:27	H&N30;Torse4;Extremity3;Perineum1;/Yes	4.2/2	33	Growth-retardation3;Hypertensionn2;Others6
Pandey et al.2009/Britain [53]	RS	1127/342:785	H&N1058;Torso 69/No	NR/1.5	1003	Growth-retardation58;Hypertension50;Moon-face58
Zhou et al.2010/China [54]	RS	23/2:21	NR/No	6/3.5	20	Cushingoid-appearance8;Poor-appetite5
Prasetyono et al.2011/Indonesia [56]	RS	749/178:571	H&N749/Yes	4.17/1.5	532	Fatiuge13;Ulceration10
Greene et al.2011/America [55]	RS	67/16:51	H&N67/No	3/2.5	56	NR
Nieuwenhuis et al.2013/Netherlands [57]	RS	21/5:16	H&N19;Torso2/No	2.5/3	13	Cushingoid-apperance8;Others4
**Study (Laser ablation)**	**Study design**	**Number of patients /Sex (M:F)**	**Location/Previous therapy**	**Age(m)/Dose(mg/kg/day)**	**Number of response**	**Complications**
Scheeper et al.1995/Scotland [58]	RS	50/8:42	H&N50/No	5.5/NR	30	Scarring 1
Chatrath et al.2002/Britain [59]	RS	36/10:26	H&N36/No	3/NR	16	Tracheocutaneous-fistula19;Scarring1
Hunzeker et al.2010/America [60]	RS	22/7:15	H&N21/No	3.45/NR	17	Hyperpigmentation2
Li et al.2010/China [61]	RS	62/23:39	NR/No	5/20J	38	Blister3;Hyperpigmentation9;Hypopigmentation3
Kaune et al.2014/German [63]	RS	38/14:24	NR/No	5/NR	25	Blister17
Su et al.2014/China [64]	RS	48/11:37	H&N20;Torso14;Extremity11;Perineum3/No	24/50J	14	Blister9;Hypopigmentation1;Scarring1
Alcántara et al. 2013/Span [62]	RS	22/2:20	H&N20;Torso1;Extremity1/No	6/NR	11	Atrophy2;Hyperpigmentation1;Ulceration1
**Study (Surgery)**	**Study design**	**Number of patients /Sex (M:F)**	**Location/Previous therapy**	**Age(m)/Dose(mg/kg/day)**	**Number of response**	**Complications**
Watanabe et al.2009/Japan [65]	RS	32/3:29	H&N26;Multiple6/Yes	15.9/NR	13	None
Kulbersh et al.2011/America [66]	RS	46/NR	H&N46/Yes	4/NR	31	Wound dehiscence1;Wound infection6
**Study (Timolol/Atenolol)**	**Study design**	**Number of patients /Sex (M:F)**	**Location/Previous therapy**	**Age(m)/Dose(mg/kg/day)**	**Number of response**	**Complications**
Semkova et al.2012/Bulgaria [69]	RS	25/10:15	NR/No	7.5/NR	4	NR
Yu et al.2013/China [71]	RS	101/NR	H&N53;Torso22;Extremity 26/No	NR/NR	57	NR
Oranje et al.2011/Netherlands [67]	RS	20/NR	H&N20/Yes	3.7/0.5	17	NR
Chan et al.2013/Sydney [68]	RCT	19/5:14	H&N12;Torse2;Extremity5 /No	2.1/0.5	15	None
Alvaro et al.2014/Chile [72]	RCT	13/6:7	NR/No	5.3/1	7	NR
Sharma et al.2013/Canada [70]	RS	22/NR	NR/Yes	3.3/NR	16	Hypotension1
Park et al.2014/Korea [73]	RS	61/NR	NR/No	NR/NR	29	None

NR, not reported; RS, retrospective study; PS, prospective study; H&N, head and neck

### Propranolol for treating IHs

**Fig 2:**
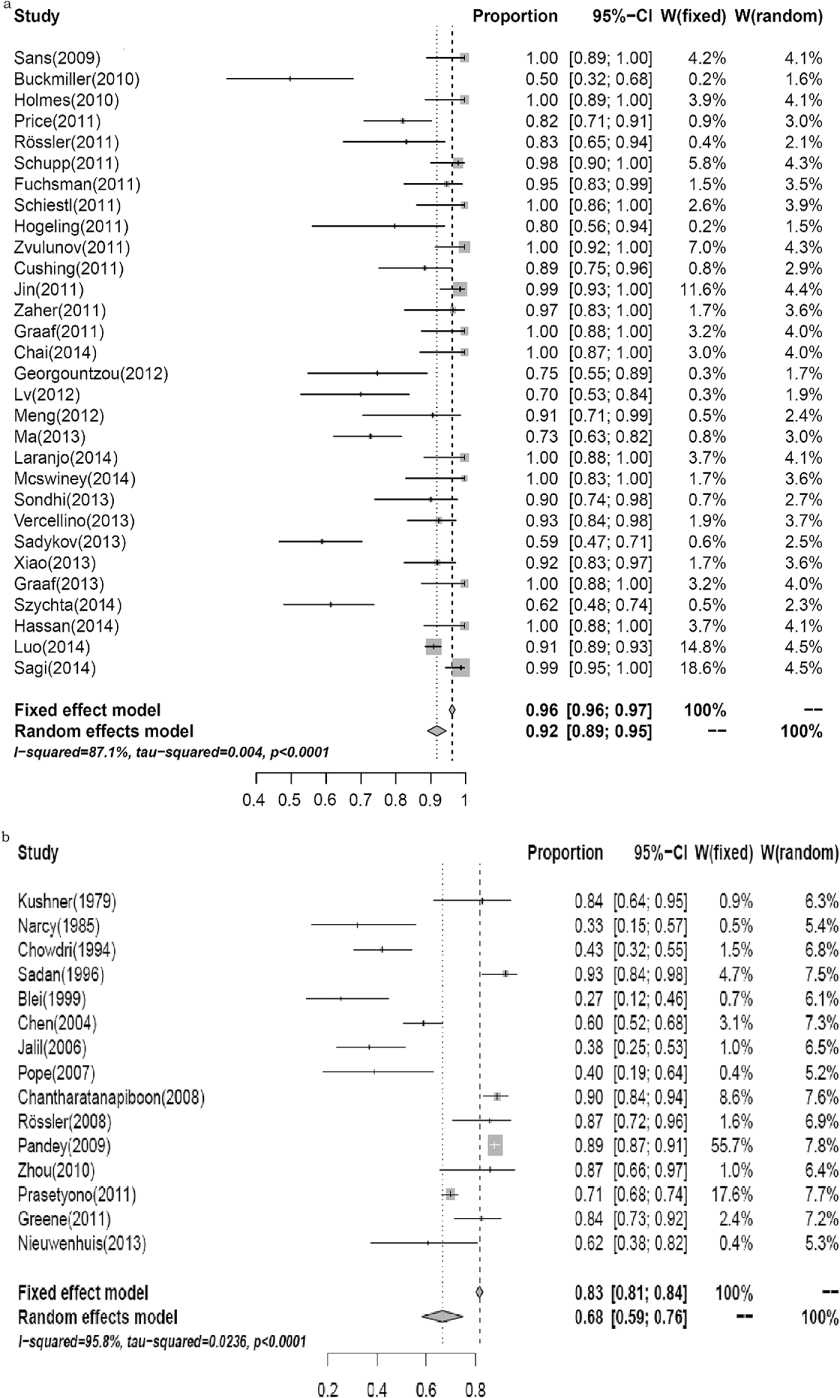
The effectiveness of propranolol (a) and systemic steroids (b) for treating IHs.

A total of 30 studies [13–42], which included 1893 individuals reported the response and side-effects of propranolol for treating IHs. The pooled odd ratio (OR) for effectiveness was 0.92 (95% CI, 0.89–0.95), and a high heterogeneity was observed between the studies (*P*_heterogeneity_ < 0.0001; *I* ^*2*^ = 87.1%) (Fig 2a). Of the included studies, 25 studies with 286 cases reported complications of propranolol treatment including hypotension (n = 33), hypoglycemia (n = 10), insomnia (n = 75), diarrhea (n = 26), and respiratory disorder (n = 28), among others (Table 3). Sensitivity analysis confirmed that excluding any of the studies from the pooled analysis did not influence the results.

**Table 3:** Complications and adverse events of propranolol (N. = 1893).

Adverse Event	No.(%)	No./N.(%)
Hypotension	33(11.54)	1.74
Hypoglycemia	10(3.50)	0.53
Insomnia	75(26.22)	3.96
Diarrhea	26(9.09)	1.37
Cold extremity	17(5.94)	0.90
Fatigue	13(4.55)	0.69
Constipation	10(3.50)	0.53
Respiratory disorder	28(9.79)	1.48
Gastrointestinal disorder	9(3.15)	0.48
Others	65(22.72)	3.43
**Total**	286(100%)	15.11%

### Subgroup analysis of propranolol for treating IHs

In the subgroup analysis, possible sources of heterogeneity such as location of the IHs, geographical distribution of the patients, mean dosage of the treatment and previous therapy (or not) were examined (Table 4). The results showed that the mean treatment dosage and previous therapy (or not) influenced the effectiveness of propranolol in treating the IHs. A propranolol dosage of 2 mg/kg/day or more resulted in better outcomes. The OR was 0.92 (95% CI, 0.88–0.95; *P*_heterogeneity_ < 0.0001; *I* ^*2*^ = 86.8%) for the 2mg/kg/day dose and 0.95 (95%CI, 0.88–1.00; *P*_heterogeneity_ < 0.0001; *I* ^*2*^ = 89%) for doses that exceeded 2 mg/kg/day; in comparison, for doses that were less than 2 mg/kg/day, the OR was 0.90 (95% CI, 0.79–1.00; *P*_heterogeneity_ < 0.001; *I* ^*2*^ = 89%). The patients with severe or intractable IHs, which did not respond to previous treatment, received subsequent oral propranolol. The effectiveness of propranolol therapy among these cases was inferior to that among the cases without previous treatment. The ORs was 0.88 (95%CI, 0.83–0.93; *P*_heterogeneity_ < 0.0001; *I* ^*2*^ = 86.5%) for the 15 studies that used some other form of treatment prior to propranolol administration; this was much lower than the OR for the 15 studies that used propranolol alone (0.95; 95%CI, 0.91–0.98; *P*_heterogeneity_ < 0.0001; *I* ^*2*^ = 88%).

**Table 4:** Stratified analysis of propranolol for treating IHs.

Stratified	No. of studies	Heterogeneity within subgroup		
		OR (95%CI)	I^2^ (%)	P for heterogeneity
**Location**:				
Head and Neck	8	0.89 (0.81, 0.97)	88.1	<0.001
Head, Neck and others	17	0.88 (0.84, 0.93)	90.4	<0.001
**Geographical location**:				
United States	7	0.86 (0.77, 0.95)	86.1	<0.001
Europe	14	0.91 (0.86, 0.96)	89.3	<0.001
Asian	9	0.96 (0.93, 0.99)	85.4	<0.001
**Mean dose(mg/kg/day)**				
< 2	4	0.90 (0.79, 1.00)	89	<0.001
= 2	21	0.92 (0.88, 0.95)	86.8	<0.001
> 2	5	0.95 (0.89, 1.00)	89	<0.001
**Prior therapy**				
Yes	15	0.88 (0.83, 0.93)	86.5	<0.001
No	15	0.95 (0.91, 0.98)	88	<0.001

### Systemic steroids for treating IHs

Fig 2b shows the results for the treatment of IHs with systemic steroids based on an analysis of 15 studies [43–57] with 2,620 participants. In the pooled analysis, the OR was 0.68 (95%CI, 0.59–0.76; *P*_heterogeneity_ < 0.0001; *I* ^*2*^ = 95.8%) for effectiveness. Sensitivity analysis showed that excluding any study from the pooled analysis did not affect the results.

### Other therapies for treating IHs

**Fig 3:**
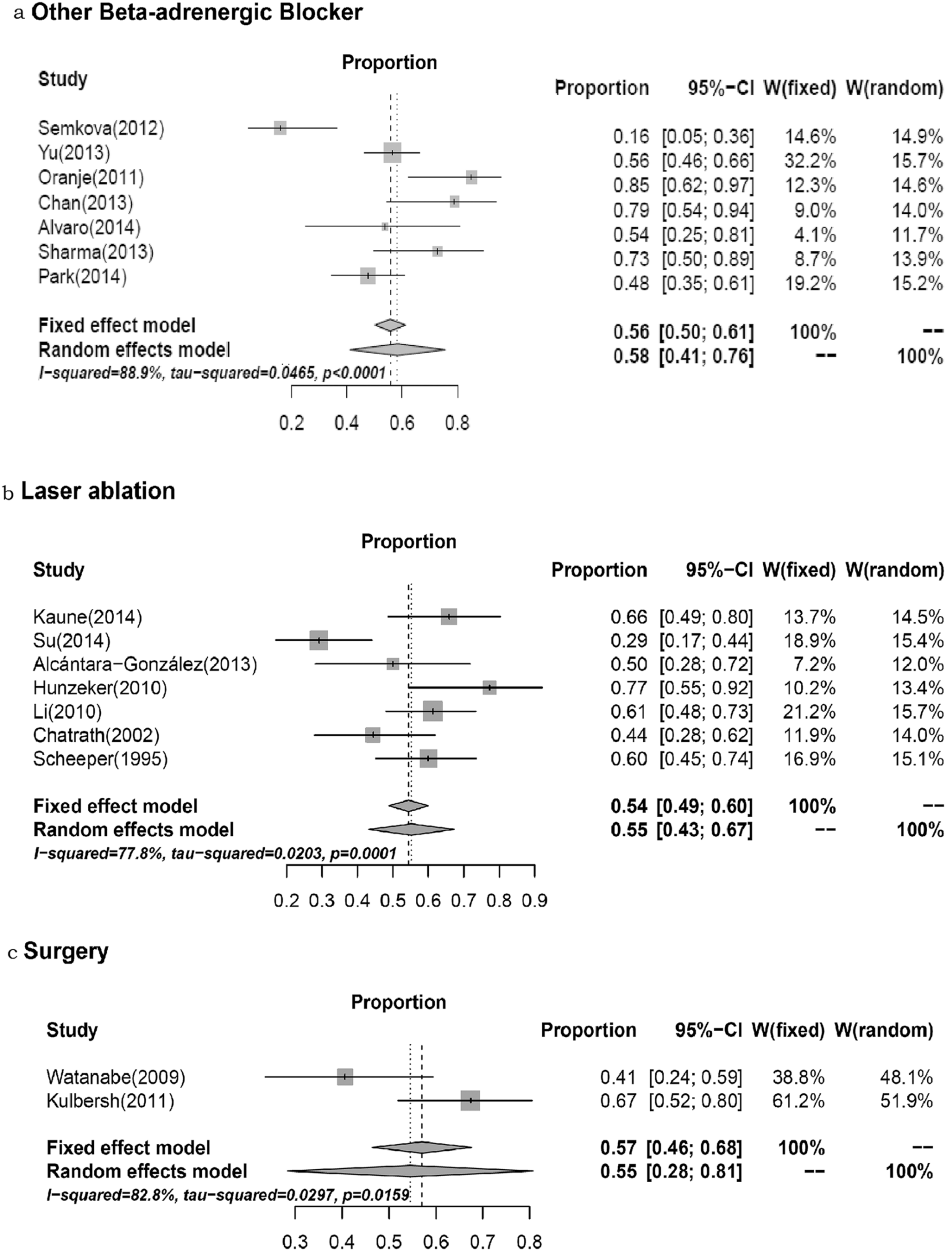
The effectiveness of other therapies for treating IHs.

Seven studies [58–64] on laser ablation, with 278 patients, were examined (Fig 3). The pooled OR for effectiveness was 0.55 (95%CI, 0.43–0.67; *P*_heterogeneity_ = 0.0001; *I* ^*2*^ = 77.8%). In addition, the OR was 0.56 (95%CI, 0.50–0.61; *P*_heterogeneity_ < 0.0001; *I* ^*2*^ = 88.9%) for the effectiveness of other beta-adrenergic blockers [67–73] and 0.55 (95%CI, 0.28–0.81; *P*_heterogeneity_ = 0.0159; *I* ^*2*^ = 82.8%) for the effectiveness of surgery [65, 66].

## Discussion

Our analysis of the 61 studies demonstrats that propranolol was more effective and safer in treating IHs than the other therapies. A subgroup analysis showed that the prefered dose of propranolol treatment was 2 mg/kg/day or more. In addition, the patients who had received previous treatments did not respond as well to propranolol treatment.

Steroids used to be the first-line treatment for IHs over the past several decades. It could be administered either locally or systemically and had a response rate of 78.05% [43–57]. Long-term steroid usage, however, tended to cause serious side effects [3]. Laser ablation, vincristine and surgical intervention have also been used to treat IHs but with varied efficiency and safety concerns [74].

Propranolol was first reported as a treatment for IHs by Lèautè-Labrère et al. in 2008 [5]. In this meta-analysis, propranolol showed a better effectiveness, with a response rate as high as 88.75%, which is 1.19 times higher than other treatments [13–42]. It is also a safer therapy, with fewer side effects [75, 76]. According to Labrèze et al., diarrhea (28/101), sleep-disorder (22/101), bronchitis (17/101) and cold hands and feet (10/101) were the common events [77]. The present study showed that propranolol treatment was more effective at a doses of 2 mg/kg/day or more [13]. However, because there is a lack in dose response studies, the optimal dose of propranolol remains to be investigated.

Recently, other beta-adrenergic blocker agents such as timolol and atenolol were reported to treating IHs. They appeared to be as effective as propranolol but with fewer side effects. Given the small number of cases reported in the literature, conclusions cannot be reached at present.

This meta-analysis is advantageous in two respects. First, a substantial number of participants were included. A meta-analysis by Peridis et al. included 13 studies, but none of them included more than 20 participants [78]. Lou et al. examined included 35 studies, but only 6 of them included more than 20 participants [79]. In this meta-analysis, 61 studies were included, and 59 of the studies had more than 20 participants. Second, data extraction, data analysis, and quality assessment were performed independently by two investigators, and consistency was achieved by a third reviewer, which enhanced the accuracy and reliability of the findings.

However, there are several limitations that should be addressed. First, the outcome measures varied across the studies, which weakened the strength of the identified association. Some of the studies used visual methods alone, while others used objective methods such as Doppler ultrasonography, MRI and endoscopy to evaluate the treatment outcomes. This discrepancy may lead to inevitable bias in the estimated ORs. Second, methodological differences among the studies may have also resulted in heterogeneity, as high *I* ^*2*^ values were observed in this meta-analysis. A subgroup analysis was performed to explore the possible heterogeneity of the studies.

Based on the findings of this analysis, a few questions remain to be answered. First, the patients with previous treatments did not respond as well to propranolol treatment. Thus, do previous IH treatments influence the effectiveness of propranolol? Second, due to the lack of dose response studies, the optimal dose of propranolol and other treatment modalities for treating IHs remains unknown. To answer these questions, further well-designed RCT studies are need to be performed.

In conclusion, propranolol is a more effective and safer treatment for IHs, and can be used as the first-line therapy for complicated IHs cases.

## Supporting Information

S1 PRISMA Checklist PRISMA 2009 Checklist. (DOC)
